# Analysis of factors associated with 1-year rebleeding in patients with acute upper gastrointestinal bleeding and a Glasgow- Blatchford Score ≥ 6 based on serological indicators

**DOI:** 10.3389/fmed.2025.1668613

**Published:** 2026-01-08

**Authors:** Li Zhang, Yuan Lan, Zheng Li Dou

**Affiliations:** Department of Gastroenterology, The Fourth Affiliated Hospital of Anhui Medical University, Hefei, China

**Keywords:** 1-year rebleeding, acute high-risk upper gastrointestinal bleeding, acute upper gastrointestinal bleeding, predictive model, GBS ≥ 6

## Abstract

**Objective:**

Analysis of serological indicators for recurrent bleeding within 1 year in patients with upper gastrointestinal bleeding and a Glasgow-Blatchford Score (GBS) ≥ 6, identification of independent risk factors, and development of a risk prediction model for recurrent bleeding within 1 year.

**Methods:**

This study enrolled 575 patients with acute upper gastrointestinal bleeding and a GBS score ≥ 6. Feature selection was performed using Lasso regression to identify statistically significant variables. The cohort was then randomly split into a training set (*n* = 400) and a validation set (*n* = 175) at a 7:3 ratio. A prediction model was developed through logistic regression analysis on the training set. Additionally, Random Forest analysis was applied, and its outcomes were visualized. The predictive performance of the newly developed model was assessed by means of a receiver operating characteristic curve, a line graph, and calibration and decision curves. Furthermore, the model was compared against the AIMS65 and GBS scoring systems to evaluate its comparative predictive value.

**Results:**

A new model was developed based on heart rate, blood pressure, hemoglobin, history of peptic ulcer, comorbid liver disease, albumin, platelet count, and CRP. The model achieved an AUC of 0.938 (95% CI: 0.915–0.961) in the training cohort, substantially higher than the AUCs for the GBS (0.590) and AIMS65 (0.562) scores. In the validation cohort, the model maintained an AUC of 0.940, compared to 0.612 for GBS and 0.637 for AIMS65. Superior performance was further evidenced by decision and calibration curve analyses, which indicated advantages in predictive accuracy, calibration, and net clinical benefit over the two conventional scores.

**Conclusion:**

The model exhibits robust performance in the prediction of 1-year rebleeding among patients scoring GBS ≥ 6, underscoring its potential clinical applicability.

## Introduction

Acute upper gastrointestinal bleeding (AUGIB) can be clinically classified into non-severe bleeding and high-risk bleeding based on disease severity (1). The characteristics of acute high-risk upper gastrointestinal bleeding include hemodynamic instability and organ dysfunction (2). Key predictors include refractory hypotension, the aspiration of bright red or coffee-ground gastric contents via nasoprognosis [be, tachycardia, and a progressive decline in hemoglobin or a level < 80 g/L]. For patients with Acute Upper Gastrointestinal Bleeding (AUGIB), the Glasgow-Blatchford Score (GBS) is commonly employed for early risk assessment and prognostication (2, 3). This scoring system incorporates parameters including pulse rate, systolic blood pressure, hemoglobin level, blood urea nitrogen (BUN), and the presence of additional clinical features (melena, syncope, liver disease, and heart failure). Studies indicate (3, 4) that a Glasgow-Blatchford Score (GBS) ≥ 6 signifies high-risk acute upper gastrointestinal bleeding (AUGIB). High-risk AUGIB typically manifests with acute onset, critical clinical presentations, and carries a high risk of mortality, posing a significant threat to patient health (2, 5). Although advancements in endoscopic techniques and acid-suppressive therapy have improved initial hemostasis success rates, rebleeding still occurs in 7–16% of patients (5–7). Rebleeding events are potentially life-threatening and fatal, constituting one of the primary prognostic indicators in AUGIB. Therefore, early prediction of rebleeding risk and heightened vigilance are crucial for significantly reducing mortality. While the Glasgow-Blatchford Score (GBS) is primarily used for early risk assessment and prognostication, there is limited clinical analysis of risk factors specifically influencing the prognosis of high-risk acute upper gastrointestinal rebleeding (defined by GBS ≥ 6), along with a paucity of more effective predictive models for this outcome (8, 9). Therefore, this study aims to investigate the independent risk factors for rebleeding within 1 year in patients with high-risk acute upper gastrointestinal bleeding (AUGIB), construct a predictive model for rebleeding, utilize machine learning for model visualization, perform internal validation using a dedicated cohort, and ultimately offer novel insights for the clinical management of this condition.

## Materials and methods

### Baseline characteristics

**FIGURE 1:**
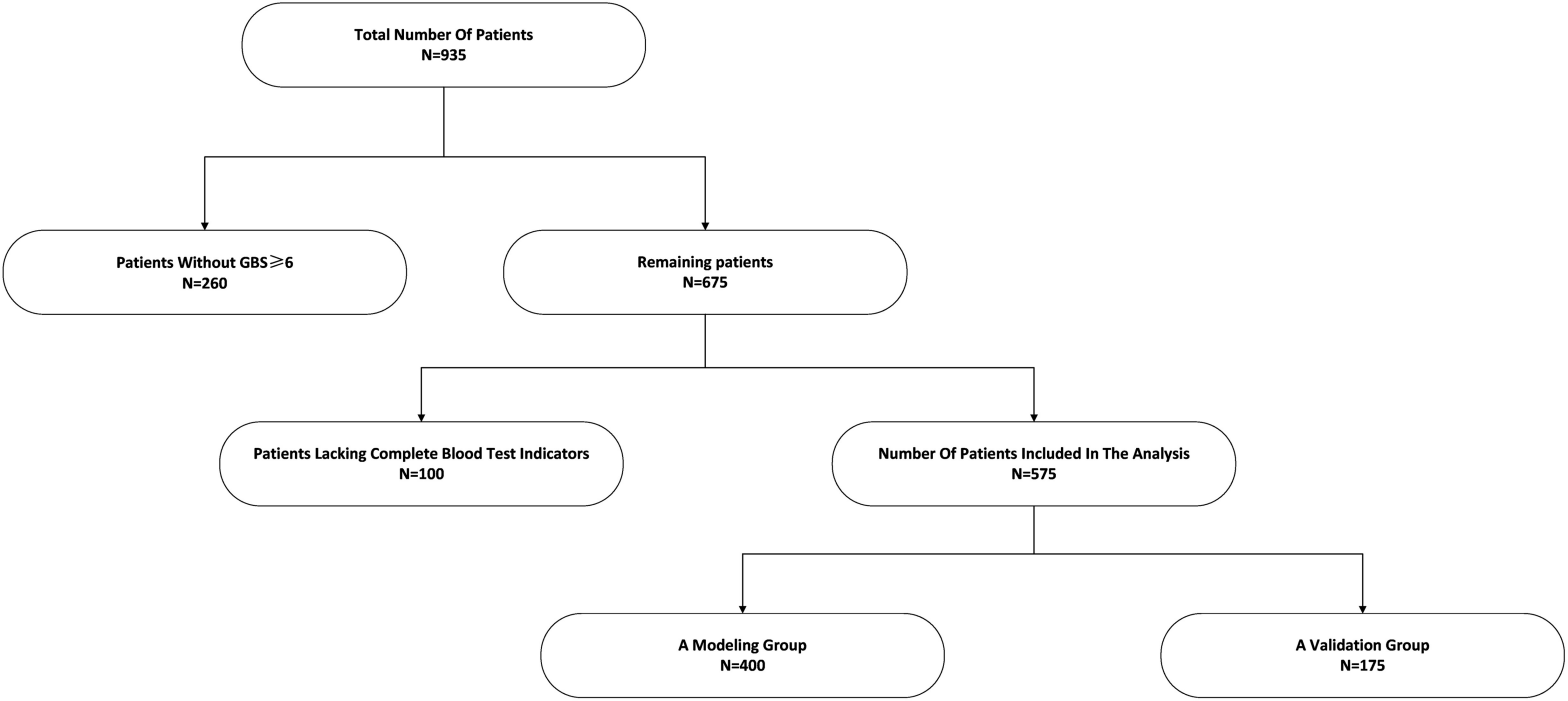
Flowchart for patients’ selection.

A retrospective analysis was conducted on 935 patients admitted with acute upper gastrointestinal bleeding (AUGIB) to the Fourth Affiliated Hospital of Anhui Medical University between January 1, 2019, and May 1, 2024. The Glasgow-Blatchford Score (GBS) was calculated for all patients, of whom 675 had a score ≥ 6. After excluding 100 cases due to incomplete clinical data, 575 patients were ultimately included in the analysis. For a visual representation, see Figure 1. Data collected for the study encompassed: Demographics and medical history: age, sex, history of peptic ulcer, liver disease, and use of NSAIDs, anticoagulants, or antiplatelet medications. Endoscopic findings: etiology (classified as 0: peptic ulcer, 1: esophageal varices; 2: Mallory-Weiss tear; 3: malignancy; 4: gastrointestinal stromal tumor) and the presence of active bleeding. Active bleeding was defined as direct visual confirmation of bleeding, manifested as spurting, oozing, visible vessel with active bleeding, or adherent clot. Hospitalization details: total length of stay and overall hospitalization cost. Clinical presentation on admission: signs and symptoms including hematemesis, melena, hematochezia, syncope, altered mental status, blood pressure, and heart rate. Laboratory parameters from the first post-admission blood test: hemoglobin (Hb), platelet count (PLT), international normalized ratio (INR), albumin (ALB), blood urea nitrogen (BUN), and creatinine (Cr). The GBS was computed for each patient according to the criteria provided in Supplementary Table 1.

### Inclusion criteria

Patients met the diagnostic standards for acute high-risk upper gastrointestinal bleeding (HR-UGIB) according to “Comparison of Glasgow-Blatchford Score and Rockall Score in Patients with Upper Gastrointestinal Bleeding” (3), defined as fulfilling all of the following: (1) Age ≥ 18 years; (2) Presence of ≥ 1 high-risk feature: Active bleeding (hematemesis, melena, or hematochezia); Circulatory failure (SBP < 90 mmHg, HR > 110 bpm); Respiratory failure (SpO2 < 90% on room air); Altered mental status; Aspiration risk; Glasgow-Blatchford Score (GBS) ≥ 6 (refer to Supplementary Table 1); (3) Clinical severity markers: Severe pallor/anemia; Persistent hematemesis or bloody stools; Syncope; Profound hypotension (SBP < 90 mmHg); Critically low hemoglobin (Hb < 80 g/L); (4) Disproportionate bleeding manifestations: Mismatch between reported blood loss (e.g., scant hematemesis) and the degree of anemia (Hb drop > 20 g/L within 24 h).

### Exclusion criteria

#### Patients were excluded from the study based on the following criteria

(1) Low-risk acute upper gastrointestinal bleeding (AUGIB), defined as a Glasgow-Blatchford Score (GBS) ≤ 1, minimal bleeding volume, and stable vital signs (systolic blood pressure ≥ 90 mmHg and heart rate < 100 beats per minute). (2) Bleeding originating from non-gastrointestinal sources, including oropharyngeal/nasopharyngeal bleeding or massive hemoptysis. (3) Pseudo-melena (drug- or diet-induced) resulting from the intake of dark-colored substances, such as animal blood products, organ meats, iron-rich foods, bismuth agents, iron supplements, or specific traditional Chinese herbs. (4) Confirmed or suspected lower gastrointestinal bleeding. (LGIB). (5) Incomplete or insufficient clinical data required for comprehensive analysis and patients who did not undergo endoscopic examination.

#### Definition of rebleeding

In this study, rebleeding was defined as recurrent active upper gastrointestinal bleeding occurring within 1 year after successful initial hemostasis (achieved through pharmacological therapy, endoscopic intervention, or combined modalities). The definition of active upper gastrointestinal bleeding is based on the “AIMS65, Glasgow-Blatchford bleeding score and modified Glasgow-Blatchford bleeding score in predicting outcomes of upper gastrointestinal bleeding: An accuracy and calibration study” (10): (1) Hematemesis or increased frequency of melena, with vomitus appearing bright red or passage of dark red bloody stools, possibly accompanied by hyperactive bowel sounds; (2) Despite rapid fluid resuscitation and blood transfusion, there is no significant improvement in the manifestations of peripheral circulatory failure, or only transient improvement followed by deterioration, with central venous pressure (CVP) remaining unstable—fluctuating or recurrently declining after brief stabilization; (3) Persistent decline in red blood cell (RBC) count, hemoglobin level, and hematocrit, accompanied by a sustained increase in reticulocyte count; (4) Persistent or recurrent elevation of blood urea nitrogen (BUN) despite adequate fluid resuscitation and urinary output; (5) Gastric tube aspirate contains significant fresh blood.

#### Grouping, modeling, and validation

A total of 575 patients with acute upper gastrointestinal bleeding and a GBS score ≥ 6 were randomly allocated to a training cohort (*n* = 400) and a validation cohort (*n* = 175) in a 7:3 ratio. Meaningful predictors were selected from the overall cohort using LASSO regression with diagnostic variable screening and variable trajectory analysis. These selected variables were subsequently incorporated into a logistic regression analysis to develop a rebleeding risk prediction model based on the training cohort. The model was visually represented using a random forest diagram. Finally, internal validation was performed using the validation cohort, demonstrating the superior performance of the new model compared to the conventional AIMS65 and GBS scores.

#### Statistical analysis

The Shapiro-Wilk test and Levene’s test were used to assess normality and homogeneity of variance, respectively. Normally distributed data are expressed as mean ± standard deviation (mean ± SD). Intergroup comparisons were performed using independent samples *t*-test when variances were equal (homogeneity of variance) and Welch’s *t*-test (*t*-test) when variances were unequal (heterogeneity of variance). Non-normally distributed measurement data are expressed as median and interquartile range (IQR). Categorical data were compared between groups using the chi-square (x^2^) test or Fisher’s exact probability test, as appropriate. Variable selection was performed within the data collection using LASSO (Least Absolute Shrinkage and Selection Operator) regression with diagnostic LASSO plots (e.g., coefficient trajectory plots). Variables selected by LASSO were subsequently entered into multivariable logistic regression analysis to construct the rebleeding risk prediction model. Model assumptions were rigorously assessed: The linearity assumption between continuous independent variables and the logit transformation of the outcome was tested using the Box-Tidwell approach. Multicollinearity among independent variables was evaluated by calculating tolerance and variance inflation factor (VIF). The model’s discriminative ability was evaluated by: Generating the receiver operating characteristic (ROC) curve. Calculating the area under the ROC curve (AUC), equivalent to the concordance statistic (C-statistic). To interpret the relative importance of predictors within the final logistic model: A Random Forest analysis was implemented using R packages. Feature (variable) importance scores were obtained and visualized. Model calibration was assessed by: Performing the Hosmer-Lemeshow goodness-of-fit test to evaluate the agreement between predicted probabilities and observed outcomes (i.e., calibration). Plotting calibration curves to visualize this agreement. Conducting decision curve analysis (DCA) and plotting the decision curve to evaluate the model’s clinical net benefit.

## Results

### Clinical characteristics of patients in the bleeding and non-rebleeding groups

Among the 575 eligible patients, 176 experienced rebleeding, while 399 did not. A history of peptic ulcer and the presence of liver disease were significantly more prevalent in the rebleeding group (*P* < 0.05). Statistically significant differences (*P* < 0.05) were observed between the two groups in heart rate, systolic blood pressure, GBS score, AIMS65 score, hemoglobin, platelet count, albumin, CRP, thrombin time (TT), prothrombin time (PT), international normalized ratio (INR), and fibrinogen (FIB) levels. Specifically, the rebleeding group exhibited significantly higher heart rates, GBS scores, AIMS65 scores, CRP levels, TT, PT, and INR, alongside lower systolic blood pressure, hemoglobin, platelet count, albumin, and fibrinogen. In contrast, no significant differences (*P* > 0.05) were found in gender, age, creatinine, blood urea nitrogen, the presence of active bleeding on endoscopy, medication history (e.g., NSAIDs, anticoagulants, antiplatelets), activated partial thromboplastin time (APTT), D-dimer, total length of stay, or total hospitalization cost. The baseline data of enrolled patients are shown in Table 1.

**TABLE 1:** Clinical characteristics of patients in the rebleeding (1) and non-rebleeding (0) groups.

Characteristics *N*	(ALL) *N* = 575	No-rebleeding *N* = 399	Rebleeding *N* = 176	*P* overall
**Gender**				0.602
Female	210 (36.52%)	149 (37.34%)	61 (34.66%)	
Male	365 (63.48%)	250 (62.66%)	115 (65.34%)	
Age	67.00 [54.00; 74.00]	66.00 [54.00; 74.00]	68.00 [52.00; 74.00]	0.94
Heart rate	85.00 [76.00; 98.00]	82.00 [75.00; 90.00]	97.00 [84.00; 103.25]	< 0.001
SBP	120.00 [106.00; 130.00]	123.00 [110.50; 132.00]	113.00 [100.75; 123.00]	< 0.001
HB	79.00 [65.00; 89.00]	84.00 [74.00; 91.00]	65.00 [57.00; 76.00]	< 0.001
BUN	9.30 [6.70; 13.75]	9.20 [6.60; 13.95]	9.65 [6.90; 13.62]	0.924
SCr	73.00 [60.00; 90.00]	74.00 [60.35; 89.00]	72.00 [59.00; 92.50]	0.663
GBS score	11.00 [9.00; 13.00]	11.00 [9.00; 13.00]	12.00 [10.00; 13.00]	< 0.001
**History of peptic ulcer disease**				< 0.001
0	448 (77.91%)	357 (89.47%)	91 (51.70%)	
1	127 (22.09%)	42 (10.53%)	85 (48.30%)	
**Liver failure**				< 0.001
0	474 (82.43%)	374 (93.73%)	100 (56.82%)	
1	101 (17.57%)	25 (6.27%)	76 (43.18%)	
**Endoscopic etiology**				< 0.001
0	409 (71.13%)	330 (82.71%)	79 (44.89%)	
1	74 (12.87%)	22 (5.51%)	52 (29.55%)	
2	22 (3.83%)	21 (5.26%)	1 (0.57%)	
3	68 (11.83%)	24 (6.02%)	44 (25.00%)	
4	2 (0.35%)	2 (0.50%)	0 (0.00%)	
**Active bleeding**				0.291
0	236 (41.04%)	170 (42.61%)	66 (37.50%)	
1	339 (58.96%)	229 (57.39%)	110 (62.50%)	
**Concomitant disease**				0.275
0	250 (43.48%)	167 (41.85%)	83 (47.16%)	
1	325 (56.52%)	232 (58.15%)	93 (52.84%)	
**Medication history**				0.022
0	393 (68.35%)	285 (71.43%)	108 (61.36%)	
1	182 (31.65%)	114 (28.57%)	68 (38.64%)	
PLT	183.00 [121.00; 218.00]	196.00 [156.00; 233.00]	118.00 [77.75; 173.25]	< 0.001
ALB	34.70 [30.40; 38.75]	35.80 [31.15; 39.40]	32.30 [29.40; 36.65]	< 0.001
CRP	0.65 [0.40; 3.42]	0.40 [0.40; 1.64]	3.34 [0.49; 8.33]	< 0.001
TT	17.50 [16.70; 18.60]	17.40 [16.70; 18.40]	17.75 [16.80; 19.02]	0.027
PT	11.70 [10.80; 13.30]	11.50 [10.70; 12.60]	12.25 [11.20; 14.30]	< 0.001
INR	1.01 [0.93; 1.11]	0.99 [0.92; 1.08]	1.05 [0.97; 1.20]	< 0.001
APTT	26.80 [23.60; 30.40]	26.80 [23.60; 30.10]	26.95 [23.80; 31.83]	0.375
FIB	2.57 [2.06; 3.38]	2.63 [2.16; 3.44]	2.43 [1.83; 3.25]	0.005
D DIMER	0.53 [0.25; 1.39]	0.48 [0.24; 1.26]	0.64 [0.28; 1.58]	0.064
Total length Stay	7.00 [6.00; 10.00]	8.00 [6.00; 10.00]	7.00 [6.00; 10.00]	0.971
Total hospitalization Cost	8,261.23 [5,972.33; 13,435.58]	8,100.13 [5,911.90; 13,453.00]	8,644.92 [6,333.99; 13,304.28]	0.226

### LASSO regression for predictor selection

**FIGURE 2:**
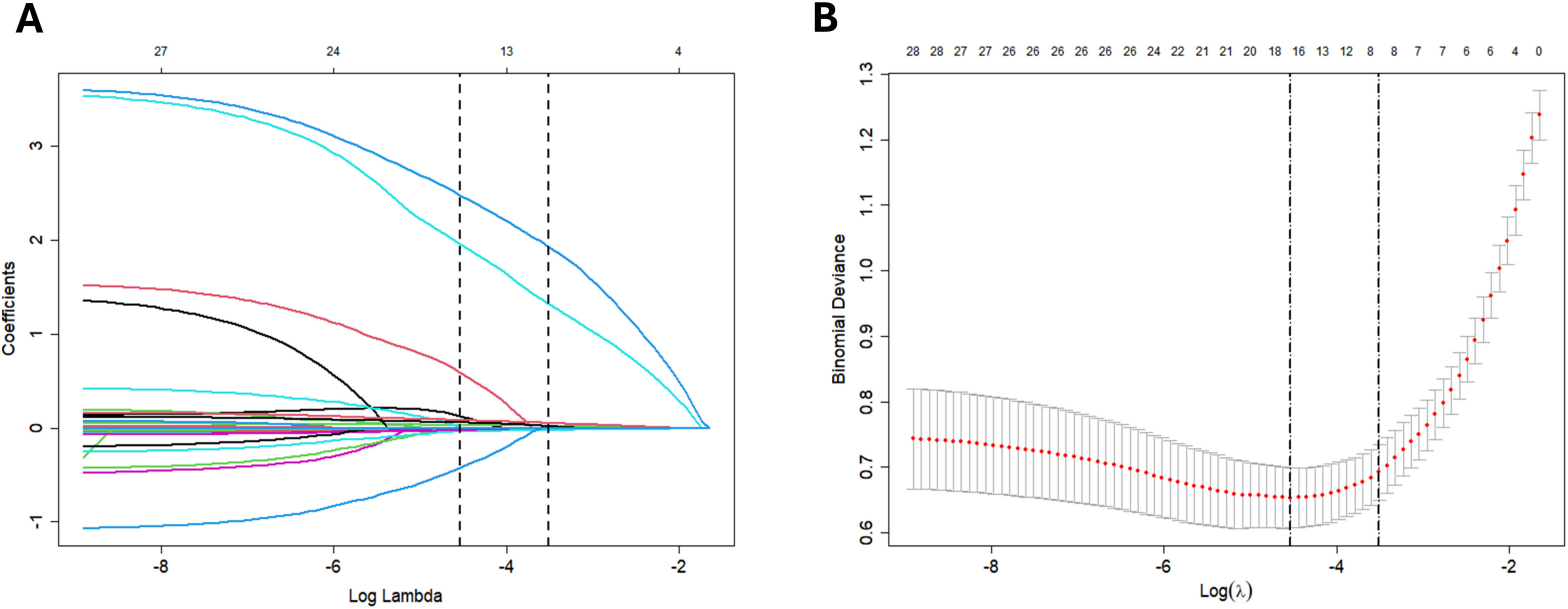
**(A)** for LASSO coefficient trajectories; **(B)** for LASSO coefficient selection.

Variables with non-zero coefficients were identified using LASSO regression with variable trajectory analysis. This process selected eight predictors: heart rate, systolic blood pressure, hemoglobin, history of peptic ulcer, history of liver disease, platelet count, albumin, and CRP. Among these, systolic blood pressure, hemoglobin, platelet count, and albumin demonstrated a negative association with 1-year rebleeding. In contrast, heart rate, history of peptic ulcer, history of liver disease, and CRP showed a positive association (Figure 2).

### Analysis of risk factors and predictive model development for 1-year rebleeding

Univariate analysis of the 400 patients in the derivation cohort identified several factors significantly associated with rebleeding (*P* < 0.05). These included a history of peptic ulcer, liver disease, an endoscopic finding of esophageal varices, as well as heart rate, systolic blood pressure, hemoglobin levels, GBS score, AIMS65 score, platelet count, and CRP levels (Table 2).

**TABLE 2:** Visualization of the univariate logistic regression analysis for factors associated with rebleeding.

Variable	Odds ratio	OR (95%CI)	*P*-value
Total hospitalization cost	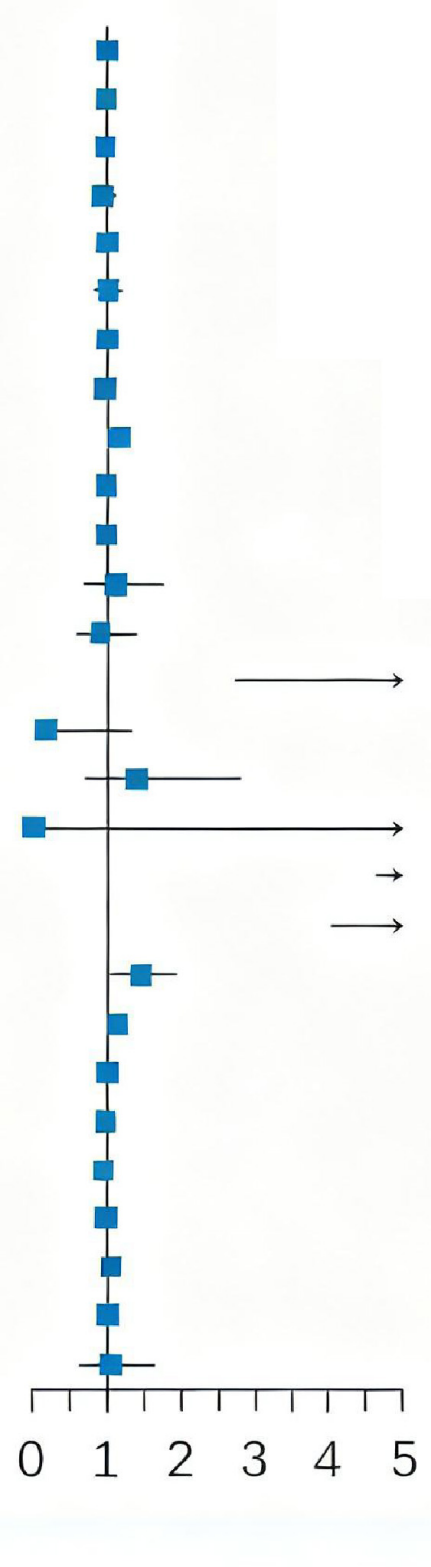	1 (1—1)	0.998
Total length stay		0.99 (0.965–1.022)	0.549
D.DIMER		0.992 (0.922–1.041)	0.778
FIB		0.934 (0.774–1.119)	0.468
APTT		1.007 (0.98–1.035)	0.589
INR		1.026 (0.826–1.234)	0.787
PT		0.998 (0.962–1.025)	0.871
TT		0.975 (0.874–1.014)	0.547
CRP		1.144 (1.091 1.207)	< 0.001
ALB		0.981 (0.951–1.011)	0.22
PLT		0.985 (0.981–0.988)	< 0.001
Medication history		1.116 (0.701–1.759)	0.64
Active bleeding		0.917 ( (0.598–1.412)	0.694
Endoscopic etiology 1		5.28 (2.81–10.24)	<0.001
Endoscopic etiology 2		0.175 (0.01–0.881)	0.094
Endoscopic etiology 3		1.403 (0.685–2.763)	0.338
Endoscopic etiology 4		0 (NA-8.801)	0.981
Liver failure		8.269 (4.732–14.90)	< 0.001
History of peptic ulcer disease		6.728 (4.098–11.22)	< 0.001
AIMS65		1.46 (1. 116–1.918)	0.006
GBS score		1.12 (1.026–1 225)	0.012
SCr		1 (0.998–1.003)	0.828
BUN		0.98 (0.944–1.016)	0.285
HB		0.943 (0.928–0.958)	< 0.001
SBP		0.966 (0.953–0.978)	< 0.001
Heart rate		1.052 (1.036–1.07)	< 0.001
Age		1.003 (0.989–1.018)	0.682
Gender		1.034 (0.662–1.629	0.883

These significant variables, selected via LASSO regression, were subsequently included in a multivariate logistic regression analysis. The results demonstrated that increased heart rate (OR = 1.036, 95% CI: 1.014–1.060, *P* < 0.01), decreased systolic blood pressure (OR = 0.969, 95% CI: 0.950–0.989, *P* < 0.01), history of peptic ulcer (OR = 19.864, 95% CI: 8.897–44.351, *P* < 0.001), presence of liver disease (OR = 9.139, 95% CI: 3.624–23.048, *P* < 0.001), decreased albumin (OR = 1.121, 95% CI: 1.058–1.187, *P* < 0.001), decreased hemoglobin (OR = 0.953, 95% CI: 0.930–0.977, *P* < 0.001), decreased platelet count (OR = 0.989, 95% CI: 0.989–0.994, *P* < 0.001), and elevated CRP (OR = 1.129, 95% CI: 1.058–1.205, *P* < 0.001) were independent risk factors for rebleeding. Multicollinearity was assessed and deemed absent, with all tolerance values > 0.1 and variance inflation factors (VIF) < 10 (Table 3).

**TABLE 3:** Visualization of the multivariable logistic regression analysis for independent predictors of rebleeding.

Characteristics	Odds Ratio (95% CI) *P*-value	
Heart rate	1.04 (1.01–1.06, *p* = 0.002)	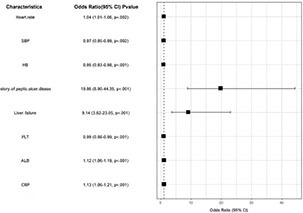
SBP	0.97 (0.95–0.99, *p* = 0.002)	
HB	0.95 (0.93–0.98, *p*< 0.001)	
Story of peptic ulcer di seas	19.86 (8.90–44.35, *p*< 0.001)	
Liver failure	9.14 (3.62–23.05, *p*< 0.001)	
PLT	0.99 (0.98–0.99, *p*< 0.001)	
ALB	1.12 (1.06–1.19, *p*< 0.001)	
CRP	1.13 (1.06–1.21, *p*< 0.001)	

The final logistic regression model was constructed as follows: Logit (P) = −0.812 + 0.036 × (Heart Rate) −0.032 × (Systolic Blood Pressure) + 2.989 × (History of Peptic Ulcer) + 2.213 × (Presence of Liver Disease) + 0.114 × (Albumin) −0.048 × (Hemoglobin) −0.011 × (Platelet Count) + 0.122 × (CRP) (Table 2). Collinearity diagnostics confirmed the absence of multicollinearity, with all variables exhibiting a tolerance > 0.1 and variance inflation factor (VIF) < 10. The constructed logistic regression model is defined by the following equation: logit (*P*) = −3.1907 + 0.053 × Heart rate + 5.248 × History of peptic ulcer + 2.38 × Concomitant liver disease + 2.61 × Concomitant malignancy − 0.0013 × Platelet count −0.059 × Hemoglobin (Hb) (Table 4).

**TABLE 4:** Multivariable logistic regression coefficients for risk prediction exported data.

		Exported data			
Characteristics	B	SE	OR	CI	*P*
(Intercept)	−0.812	2.01009	0.444	0.444 (0.009–22.816)	0.686
Heart rate	0.036	0.01134	1.036	1.036 (1.014–1.060)	< 0.01
SBP	−0.032	0.01026	0.969	0.969 (0.950–0.989)	< 0.01
HB	−0.048	0.01238	0.953	0.953 (0.930–0.977)	< 0.001
History of peptic ulcer disease	2.989	0.4098	19.864	19.864 (8.897–44.351)	< 0.001
Liver failure	2.213	0.47194	9.139	9.139 (3.624–23.048)	< 0.001
PLT	−0.011	0.00303	0.989	0.989 (0.983–0.994)	< 0.001
ALB	0.114	0.02937	1.121	1.121 (1.058–1.187)	< 0.001
CRP	0.122	0.03323	1.129	1.129 (1.058–1.205)	< 0.001

### Present a visual representation of the machine learning model

**FIGURE 3:**
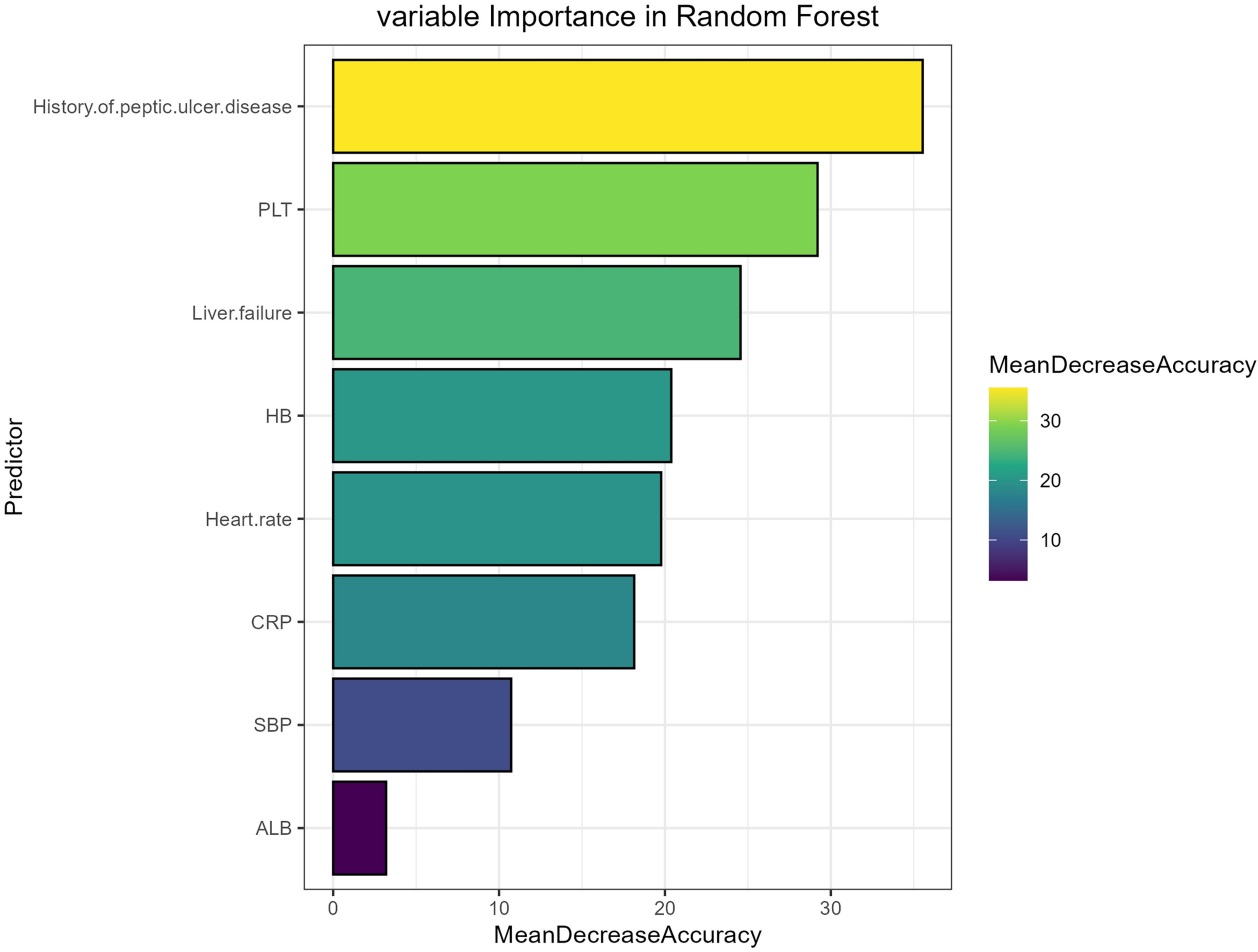
Random Forest feature importance plot.

The independent risk factors (history of ulcer disease, HB, PLT, ALB, heart rate, SBP, liver failure, CRP) screened-out by the multivariate logistic risk regression model were ranked according to their importance by machine learning using R language. The ranking assigned by the new prediction model in the order of high to low was as follows: history of ulcer disease > PLT > Liver failure > HB > Heart rate > CRP > SBP > ALB. Thus, as shown in Figure 3. The most important risk factor in the prediction model was history of ulcer disease, followed by PLT, Liver failure (Figure 3).

### Comparison of predictive performance between the training and validation groups based on the predictive factors

**FIGURE 4:**
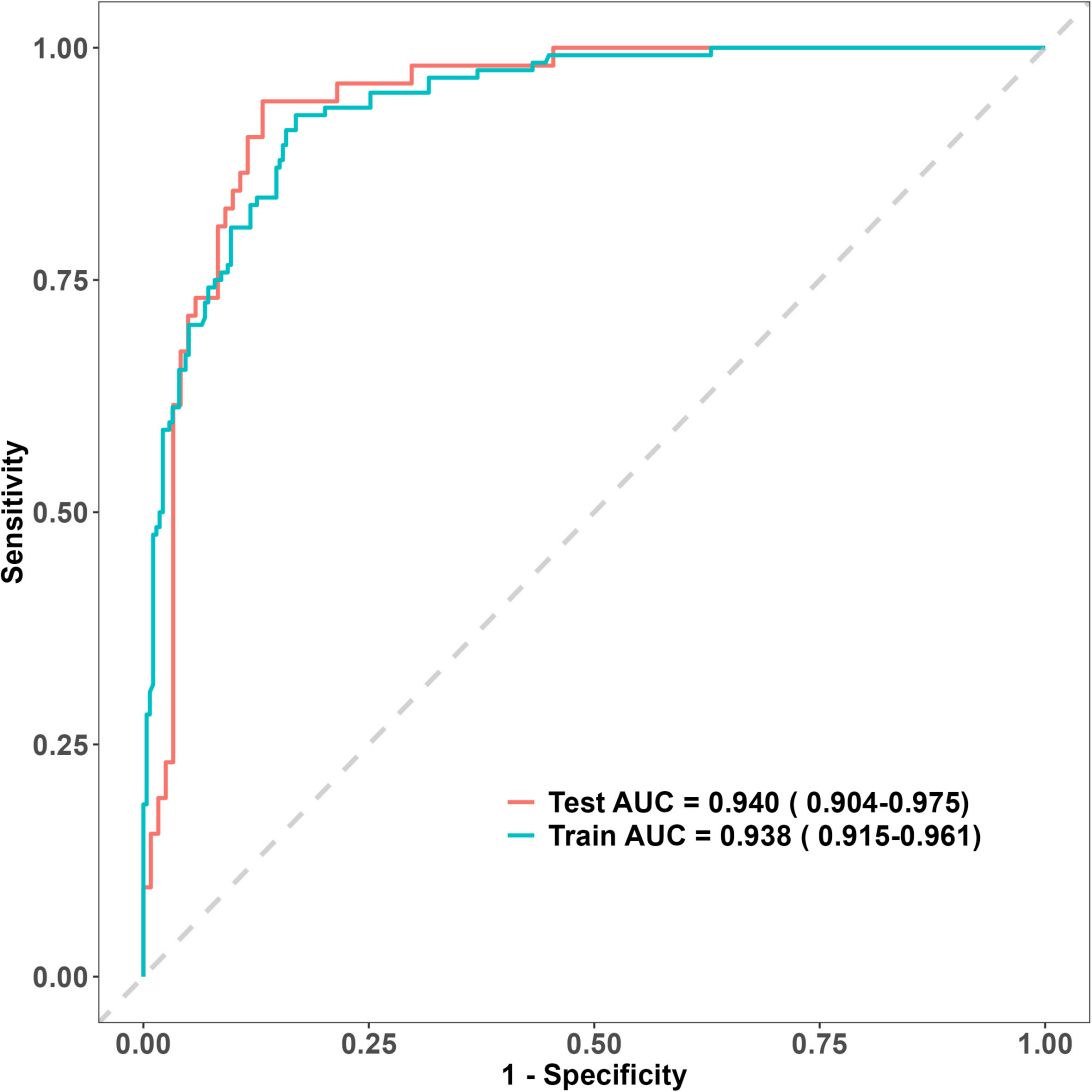
ROC curves for the training and validation cohorts.

**FIGURE 5:**
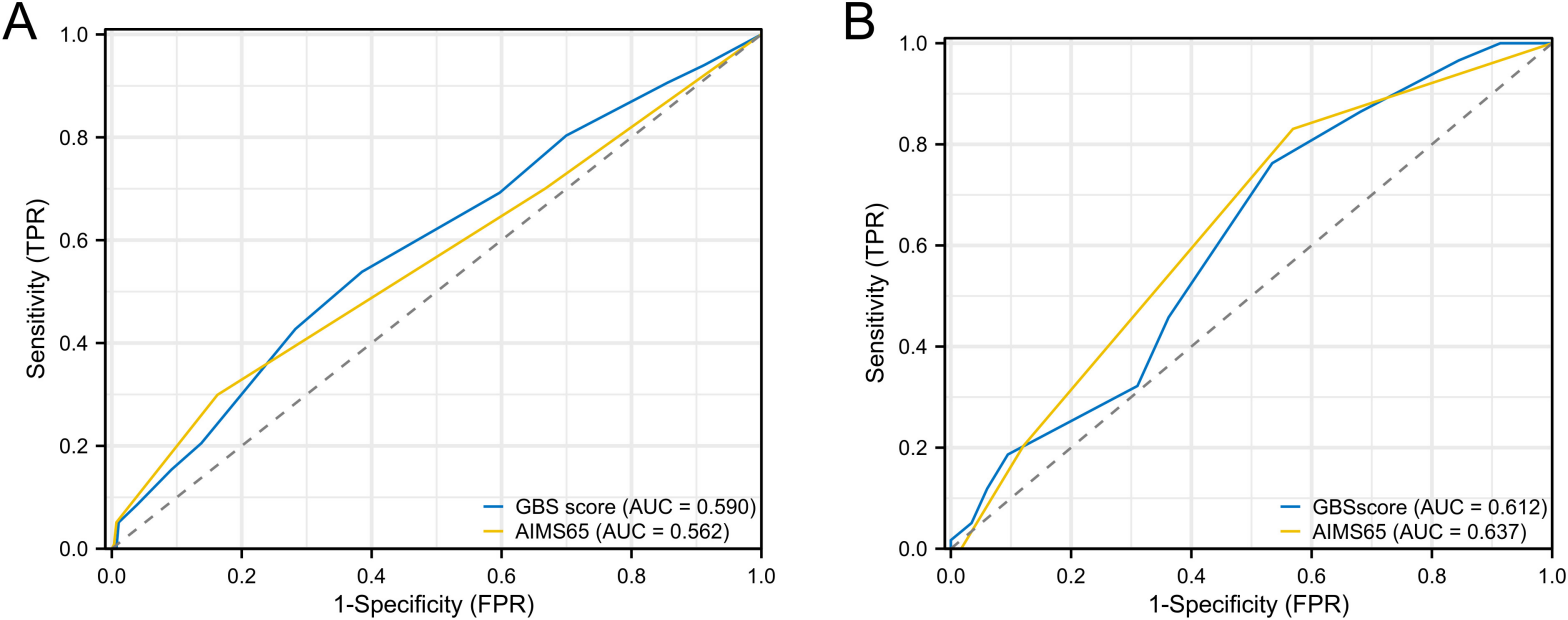
**(A)** shows the C-indices of the GBS and AIMS65 scores in the training cohort; **(B)** shows the C-indices of the GBS and AIMS65 scores in the validation cohort.

The predictive model demonstrated excellent discriminatory ability, with an AUC of 0.938 (95% CI: 0.9153–0.9612) in ROC analysis, sensitivity of 83.1%, and specificity of 92.7%. Internal validation using the validation cohort (*n* = 175) demonstrated robust model performance. Predicted probabilities derived from the logistic regression equation yielded an AUC of 0.940 (95% CI: 0.9041–0.9751) on ROC analysis, with 86.8% sensitivity and 94.2% specificity (Figure 4). The AUC value of the new predictive model was compared with those of the conventional GBS and AIMS65 scores. In the training cohort, the predictive accuracy (AUC) of the new model was 0.938, compared with 0.590 for the GBS score and 0.562 for the AIMS65 score. In the validation cohort, the corresponding AUC values were 0.940 for the new model, 0.612 for the GBS score, and 0.637 for the AIMS65 score. DeLong’s test revealed statistically significant differences in AUC values between the new model and both the GBS score and AIMS65 score (*P* < 0.05), indicating that the new predictive model exhibits superior predictive performance compared to the GBS and AIMS65 scores (Figure 5).

**FIGURE 6:**
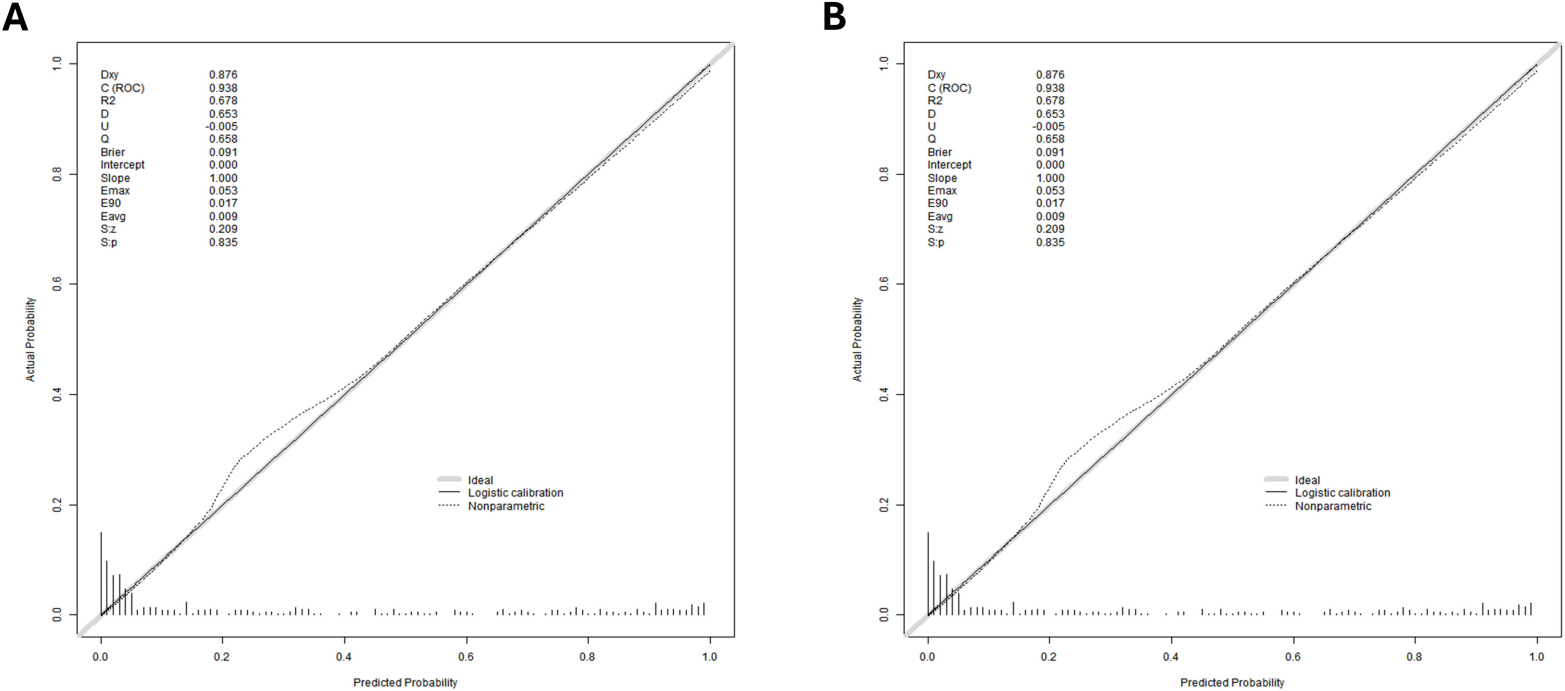
**(A)** for a model training cohort; **(B)** for a validation cohort.

In the training cohort, the Hosmer-Lemeshow goodness-of-fit test indicated adequate calibration (χ^2^ = 5.9602, *P* = 0.6517). For the validation cohort, the test yielded χ^2^ = 60.717, *P*< 0.01). These results demonstrate that the predictive model exhibits satisfactory discriminatory ability and calibration performance (Figure 6).

**FIGURE 7:**
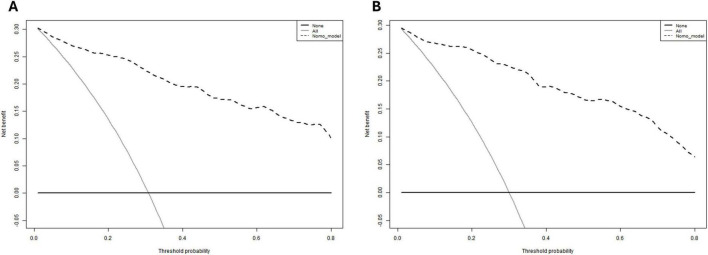
**(A)** for a model training cohort; **(B)** for a validation cohort.

**FIGURE 8:**
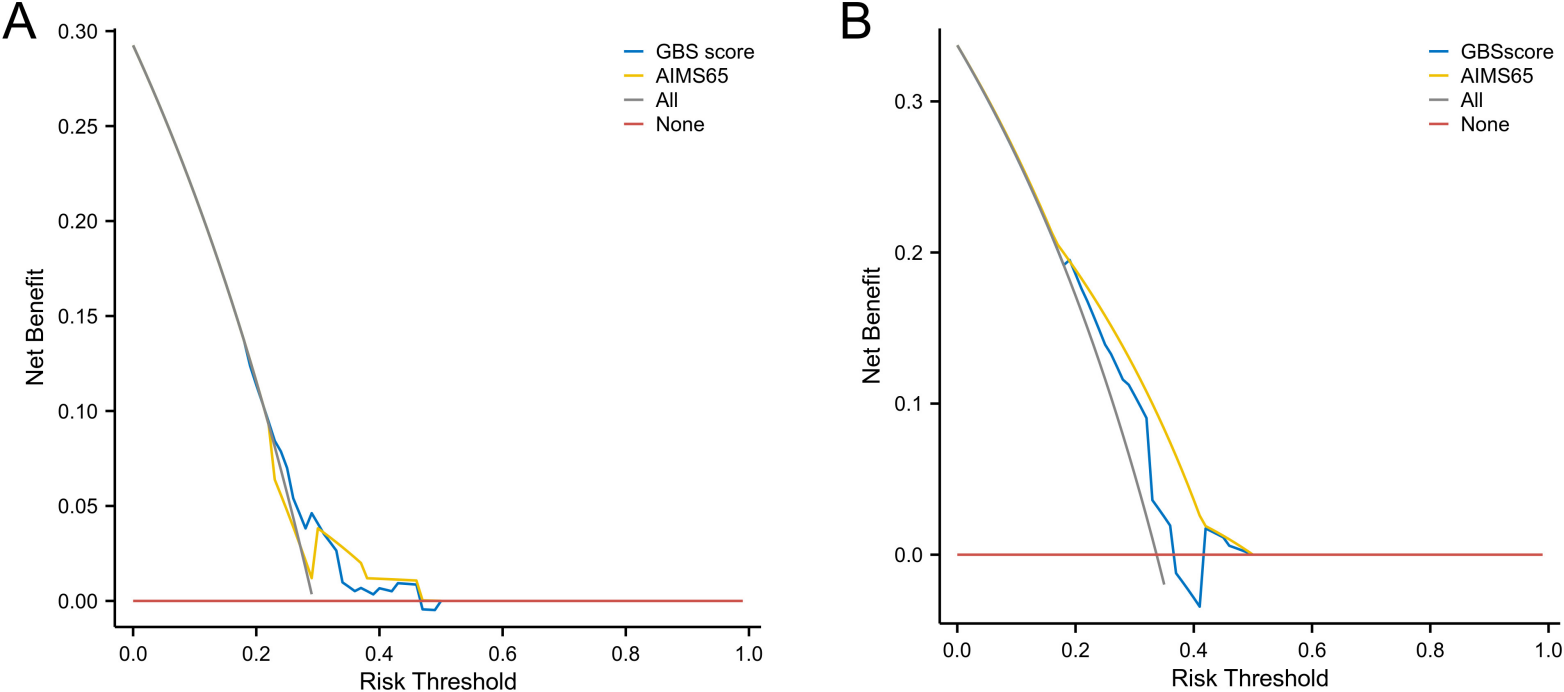
**(A)** The decision curve analysis (DCA) of the GBS and AIMS65 scores in the training cohort. **(B)** The decision curve analysis (DCA) of the GBS and AIMS65 scores in the validation cohort.

As demonstrated in Figure 7, clinical decision-making guided by this model yields significantly higher net benefit. The model shows strong agreement between predicted and observed outcomes in both training and validation cohorts, confirming its robust predictive accuracy, consistency, and clinical utility. Decision curve analysis further confirmed that our predictive model demonstrated superior net benefit across most of the reasonable threshold probabilities in both the training and validation cohorts, compared to the GBS and AIMS65 scoring systems (Figure 8).

## Discussion

Acute high-risk upper gastrointestinal bleeding (UGIB) is associated with a high rate of rebleeding, which serves as a critical risk factor for mortality. Therefore, accurate prediction of rebleeding risk and enhanced preventive measures can help reduce both rebleeding incidence and mortality, making it an essential component of emergency management strategies (5, 11, 12). Current evidence on rebleeding predictors predominantly concerns ANVUGIB and variceal bleeding, leaving a knowledge gap in risk stratification tools specifically designed for acute high-risk UGIB cases (5, 13, 14) The aging population has led to an increasing proportion of elderly patients with acute high-risk UGIB, who often present with multiple comorbidities and declining organ function. Concurrently, the growing use of anticoagulants, antiplatelet agents, and NSAIDs has further complicated clinical management. These combined factors make reducing rebleeding rates in this vulnerable population a critical challenge in contemporary clinical practice.

Using a 5-year retrospective cohort design, we systematically evaluated hemodynamic parameters, clinical presentations, and laboratory biomarkers in eligible acute high-risk UGIB patients to elucidate predictors of rebleeding. The analysis identified a history of peptic ulcer, hemoglobin (Hb), platelet count (PLT), albumin (ALB), heart rate, systolic blood pressure (SBP), liver disease, and CRP as significant independent risk factors for 1-year rebleeding in patients with acute upper gastrointestinal bleeding. Based on these predictors, we developed a novel predictive model that demonstrates superior rebleeding risk stratification, offering clinically actionable insights for patient management. Hepatic dysfunction leads to decreased production and impaired functionality of clotting factors, resulting in substantial disruption of physiological coagulation (12, 13). Patients with cirrhosis demonstrate significant coagulation abnormalities, including elevated plasma fibrinogen levels, increased platelet activation and heightened plasminogen activator activity, collectively contributing to systemic anticoagulation imbalance (15). Acute high-risk UGIB secondary to esophagogastric variceal rupture in decompensated cirrhosis with portal hypertension represents a life-threatening complication, characterized by sudden onset, high rebleeding rates, and significant mortality—substantially amplifying patient fatality risks (16). Hemodynamic instability is a critical risk factor for rebleeding and mortality, typically manifested by systolic blood pressure (SBP) < 90 mmHg, heart rate > 100 bpm and signs of shock (17). Our findings are consistent with this conclusion.

During the early stages of rebleeding, hemoglobin (Hb) levels may remain relatively stable due to compensatory physiological mechanisms, including peripheral vasoconstriction and redistribution of circulating erythrocytes. However, as the condition progresses, substantial transudation of interstitial fluid into the vascular compartment occurs to restore blood volume, resulting in hemodilution and a subsequent decline in measured Hb concentrations (18). The resultant hemodilution-induced decline in hemoglobin (Hb) levels impairs systemic oxygen delivery, leading to tissue hypoxia that particularly compromises gastrointestinal mucosal repair mechanisms, ultimately worsening clinical outcomes. Consistent with the findings of some foreign literatures (5, 19), our study also identified decreased hemoglobin (Hb) levels as an independent risk factor for acute UGIB, reinforcing the clinical relevance of anemia in risk stratification. In this study, elevated CRP levels were associated with an increased risk of rebleeding. This association may be attributed to the fact that elevated CRP reflects systemic or local inflammatory activity, which can exacerbate mucosal injury, predispose to infection, disrupt coagulation, and impede mucosal healing—all pathways that collectively increase the likelihood of rebleeding. In this study, a history of peptic ulcer disease significantly increased 1-year rebleeding risk. The underlying mechanism likely involves incomplete healing of mucosal defects at the original bleeding site. When re-exposed to gastric acid and digestive enzymes, these vulnerable lesions are prone to recurrent hemorrhage. Serum albumin (ALB) serves as both an indicator of nutritional status and a key inflammatory biomarker, playing a critical role in disease progression, including malignancies. Albumin serves not only as the primary regulator of plasma colloid osmotic pressure but also exerts critical anti-inflammatory, antioxidant, and endothelial-stabilizing effects, in addition to its role in maintaining coagulation-anticoagulation balance. A significant decrease in its levels fosters a systemic and local environment that is profoundly unfavorable for hemostasis and tissue repair. Our study identify hypoalbuminemia as a statistically independent risk factor for rebleeding, existing evidence consistently demonstrates (6, 20) that low serum albumin (ALB) independently predicts rebleeding and mortality.

Previous studies have identified advanced age as an independent risk factor for rebleeding, primarily due to elderly patients’ higher comorbidity burden, declining organ function, and poorer prognosis (17). However, our study did not demonstrate age as a statistically significant predictor of rebleeding, potentially attributable to limited sample size in the elderly subgroup. In clinical practice, heightened vigilance remains essential for elderly patients—particularly those with multiple comorbidities—to mitigate risks of rebleeding and mortality, despite the non-significant statistical association in our study. The primary role of PLTs is coagulation and hemostasis. The PLT membrane is attached to a plasma layer (the outer covering of PLTs) composed of plasma proteins, coagulation factors, and molecules related to the fibrinolytic system; this membrane plays a role in the hemostasis process after vascular injury. A notable observation is that if the patient’s thrombocytopenia results in prolonged bleeding time, severe injury or rebleeding can occur under stress (21), in the present study, a lower platelet count was associated with a higher incidence of rebleeding. The novelty of this study lies in: 1. Innovation in Research Subjects: Instead of categorizing patients by the conventional etiology-based classification (variceal vs. non-variceal) (22–24), as abundant literature already exists on this topic (13, 25–27), we selected cases based on a Glasgow-Blatchford Score (GBS) ≥ 6 for investigation. This approach focuses on a “blind spot” in clinical practice—specifically, our study is the first to systematically develop and validate a prediction model tailored to this distinct high-risk population (GBS ≥ 6). Rather than merely replicating existing research, we aim to address a critical gap in current clinical practice by providing a tailor-made, precision risk-stratification tool for high-risk UGIB patients (28–30); 2. Innovation in Predictor Variables: We introduced novel biomarkers, incorporating inflammatory (C-reactive protein) and nutritional (albumin) markers (27). Statistical results confirmed that both are independent risk factors for rebleeding. This represents not merely an expansion of variables but a paradigm shift in modeling philosophy: moving from “describing symptoms” to “addressing underlying biological processes,” thereby providing a more robust scientific foundation for prognostic assessment; 3. Substantial Methodological and Performance Advantages: We implemented a modern modeling pipeline combining LASSO regression (ensuring robust variable selection) with logistic regression (maintaining model interpretability), supplemented by random forest algorithm for validation and visualization. This rigorous methodology ensures model reliability and differs fundamentally from statistical approaches employed in previous studies (31–34). Although this predictive model demonstrates certain prognostic value for 1-year rebleeding risk in acute high-risk UGIB patients, the study has inherent limitations as a single-center retrospective analysis. With technological advances and heightened health awareness, the incidence of acute upper gastrointestinal bleeding (AUGIB) patients with Glasgow-Blatchford Score (GBS) ≥ 6 has progressively declined, accompanied by reduced rebleeding rates within 1 year. Consequently, the limited sample size and single-center origin of this study may introduce confounding factors affecting the results, representing notable methodological limitations. The current study lacks external validation of the constructed rebleeding prediction model. Therefore, widespread clinical application of this model to all acute high-risk UGIB patients requires further rigorous multicenter prospective studies to confirm its predictive accuracy and generalizability.

## Conclusion

Elevated heart rate, decreased systolic blood pressure, low hemoglobin levels, a history of peptic ulcer, comorbid liver disease, hypoalbuminemia, thrombocytopenia, and elevated CRP were identified as independent risk factors for 1-year rebleeding in patients with acute upper gastrointestinal bleeding. Developing and validating a logistic regression model can effectively predict the occurrence of rebleeding within 1 year in patients, enabling clinicians to identify high-risk patients for targeted early interventions.

## Data Availability

The original contributions presented in the study are included in the article/Supplementary material, further inquiries can be directed to the corresponding author.
