# Supplementary figures and images for: Analysis of factors associated with 1-year rebleeding in patients with acute upper gastrointestinal bleeding and a Glasgow- Blatchford Score ≥ 6 based on serological indicators
Source: Front Med (Lausanne). 2026 Jan 8;12:1668613. doi: 10.3389/fmed.2025.1668613 (PMC12823913; doi:10.3389/fmed.2025.1668613)

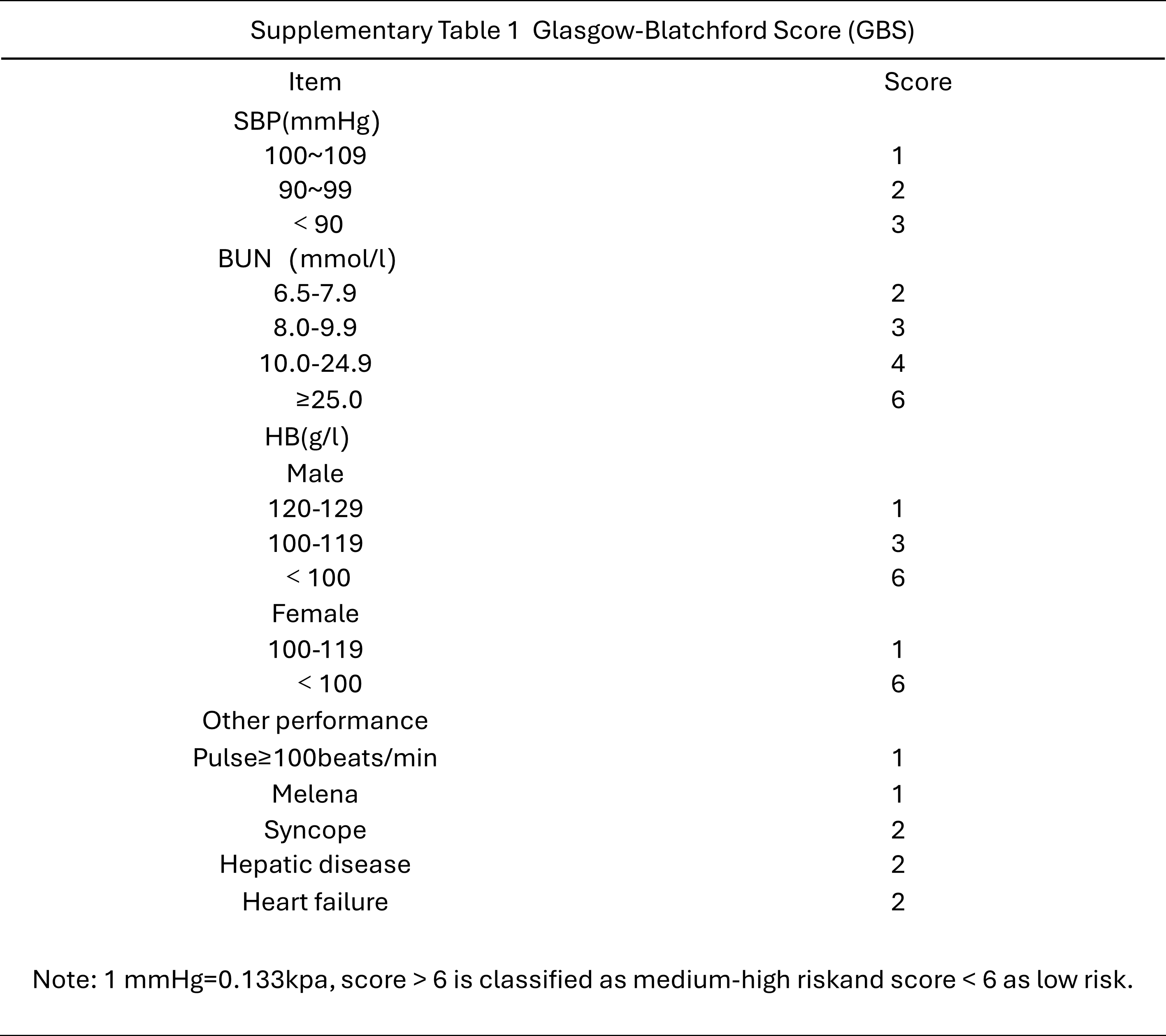

Supplement: Supplementary file 1 [file Image_1.tif]
